# Composition and Nutritional Value of Acid Oils and Fatty Acid Distillates Used in Animal Feeding

**DOI:** 10.3390/ani11010196

**Published:** 2021-01-15

**Authors:** Elisa Varona, Alba Tres, Magdalena Rafecas, Stefania Vichi, Ana C. Barroeta, Francesc Guardiola

**Affiliations:** 1 Departament de Nutrició, Ciències de l’Alimentació i Gastronomia, Campus de l’Alimentació de Torribera, Facultat de Farmàcia i Ciències de l’Alimentació, Universitat de Barcelona, Av Prat de la Riba, 08921 Santa Coloma de Gramenet, Spain; evarona@ub.edu (E.V.); stefaniavichi@ub.edu (S.V.); fguardiola@ub.edu (F.G.); 2 Institut de Recerca en Nutrició i Seguretat Alimentària (INSA-UB), Universitat de Barcelona, Av Prat de la Riba, 08921 Santa Coloma de Gramenet, Spain; magdarafecas@ub.edu; 3 Departament de Nutrició, Ciències de l’Alimentació i Gastronomia, Campus Diagonal, Facultat de Farmàcia i Ciències de l’Alimentació, Universitat de Barcelona, Av. de Joan XXIII, 08028 Barcelona, Spain; 4 Animal Nutrition and Welfare Service (SNiBA), Animal and Food Science Department, Faculty of Veterinary, Universitat Autònoma de Barcelona, Edifici V, Travessera dels Turons, 08193 Bellaterra, Spain; Ana.Barroeta@uab.cat

**Keywords:** fat by-products, acid oils, fatty acid distillates, animal feed, nutritional value, poultry, pig, MIU value, energy

## Abstract

**Simple Summary:**

Acid oils and fatty acid distillates are by-products from the edible oil refining industry that are rich in free fatty acids. Their use as feed ingredients is a way to valorize them in order to increase the sustainability of the food chain; however, differences in the animal productive parameters when using them have been reported. The objective of this study is their characterization and the identification of their sources of variability. Results have revealed a high variability in their composition, being influenced both by the botanical origin of de crude oil and by the type of refining process. Thus, the analytical control and standardization of these by-products is of outmost importance to guarantee a standardized quality which would increase their value as feed ingredients. Remarkably, almost all samples showed some compositional values above the limits recommended by some feed fat guidelines, which suggests that the production of these by-products must be standardized and improved, and some of the thresholds should probably be revised.

**Abstract:**

Acid oils (AO) and fatty acid distillates (FAD) are oil refining by-products rich in free fatty acids. The objective of this study is their characterization and the identification of their sources of variability so that they can be standardized to improve their use as feed ingredients. Samples (n=92) were collected from the Spanish market and the MIU value (sum of moisture, insoluble impurities, and unsaponifiable matter), lipid classes, fatty acid composition, and tocol content were analyzed. Their composition was highly variable even between batches from the same producer. As FAD originated from a distillation step, they showed higher free fatty acid amounts (82.5 vs 57.0 g/100 g, median values), whereas AO maintained higher proportions of moisture, polymers, tri-, di-, and monoacylglycerols. Overall, the MIU value was higher in AO (2.60–18.50 g/100 g in AO vs 0.63-10.44 g/100 g in FAD), with most of the contents of insoluble impurities being higher than those in the guidelines. Tocol and fatty acid composition were influenced by the crude oil’s botanical origin. The calculated dietary energy values were, in general, higher for AO and decreased when a MIU correction factor was applied. The analytical control and standardization of these by-products is of the outmost importance to revalorize them as feed ingredients.

## 1. Introduction

Many vegetable crude fats and oils need to be refined to agree with the established parameters in the regulations according to their intended use, to increase their shelf life and consumer acceptance [1]. Essentially, refining consists of removing free fatty acids (FFA) and other non-desirable components from crude oils, either by chemical or physical processes. Usually, chemical refining comprises various main steps, namely degumming, neutralization (when FFA are removed), winterization (optional), bleaching, and deodorization. The main steps of a physical refining process usually are degumming, winterization (optional), bleaching, and deodorization [2]. However, despite the well-controlled refining procedures, some components with positive effects such as essential fatty acids (FA), tocopherols (T), polyphenols, or sterols are concomitantly removed from crude oils and accumulated in the by-products. Acid oils (AO) from chemical refining and fatty acid distillates (FAD) from physical refining are by-products obtained from the refining steps where the FFA removal mainly takes place: (i) In the chemical refining, FFA are removed in the neutralization step in which an alkali (usually sodium hydroxide) is added to the degummed oil to precipitate FFA as soap-stocks that are then removed by centrifugation and acidulated to obtain AO; (ii) in physical refining, FFA are mainly removed by distillation in the deodorization step when oil is subjected to steam stripping at high temperatures under vacuum, with FAD being the by-product of this distillation [2,3]. Consequently, FFA are the major components in both AO and FAD and this provides them a high energetic value. They contain other compounds, such as lipid soluble vitamins, that are also removed during these refining steps. Because of this composition, they are valuable products for certain uses, and by this, they do not become waste products and harmful to the environment [4]. They present a growing interest in animal feeding, which is an important application to upcycle and valorize them [5,6]. Indeed, both AO and FAD are included in the European Catalogue of feed materials that shall be of voluntary use by the feed business operators [7]. Since AO and FAD are fat products, supplementing animal feeds with them would be a way to supply energy and fat-soluble vitamins to the animal diet [6,8].

The energy value of a fat depends on its quality and composition, as well as on the species, gender, and age of the animal [9]. Fat quality is often understood, simplified and in a practical way, as the amount of compounds that can dilute the energy content of fats, such as moisture and other volatile compounds (M), insoluble impurities (I), and unsaponifiable matter (U) that are globally known as the MIU value. The non-elutable material value (NEM) is another quality measure of fat that might contain M, I, U, and the oxidized and polymerized lipids [9]. According to previous studies, AO could be considered as potential cheap energy ingredients in poultry diets, as long as they maintain a minimum quality (pH ≥ 5 and low MIU content) [10,11]. However, AO and FAD quality is not always controlled or reported, nor are limits found for all these parameters in all feed fat regulations or guidelines. As an example, the European regulation states that M has to be reported in AO and FAD if it exceeds 1 g/100 g [7]. Some guidelines from associations for the development of animal feeding strategies state that I and MIU should be below 0.15 and 5 g/100 g, respectively, both for AO and FAD [12].

On the other hand, the fat composition, and the species, gender, and age of the animal, influence fat digestion and absorption, which are the main factors affecting the energy that animals can obtain from a fat. In this respect, the most relevant points of fat composition are the FFA content and the FA composition, especially the length of the FA carbon chain and the degree of saturation that are related to the oil source [13,14]. High FFA contents have been associated with digestibility impairments especially for saturated fats and for certain animal species and ages [15,16,17]. However, even if AO and FAD are rich in FFA, they have been suggested as valuable fat sources for feed [17,18].

However, there is a lack of characterization of AO and FAD, and to achieve it, analytical methods adapted to these particular products need to be used [19]. This means that detailed and representative information on their composition is scarcely present in the feed ingredient composition tables [12,20]. Moreover, in studies that have dealt with some AO and FAD, their quality and composition have been reported to be very variable [4]. This is one of the reasons that, nowadays, many feed producers and farmers are reluctant to use them routinely. In many cases, they even encounter differences in the productive parameters between batches from the same AO or FAD producer. Therefore, determining the compositional parameters and the variability of these by-products, which is related to their nutritional value, is essential for the compliance of the minimum composition and quality requirements in terms of raw materials for animal feeding. Our hypothesis is that both the botanical origin of the crude oils as well as the refining process might affect the final AO and FAD composition, and that some compositional parameters might be more affected, leading to differences in the nutritional value of these by-products. Thus, the objective of this paper is to characterize AO and FAD available in the Spanish market, to detect and evaluate their sources of variability especially focusing on the parameters that determine the nutritional value of fat products, and to establish recommendations on the control of these parameters.

## 2. Materials and Methods

### 2.1. Samples

For this study, a total of 92 FFA-rich by-products of edible oil refining and intended for animal feeding were collected from the Spanish market: 79 samples were by-products from chemical refining (AO) and 13 from physical refining (FAD). The samples were obtained from edible oil refineries, AO producers (companies that buy soap-stocks to refineries and produce AO), and feed producers. Mostly, the samples were blends coming from the refining of different edible fats and oils, and only 43 samples came from a single fat or oil, reflecting the usual availability of these products (Table 1).

Once samples arrived at the laboratory, they were melted, homogenized, and divided into smaller vials. Their head space was filled with N_2_ and samples were stored at −20 °C until analysis. The melting conditions varied according to the botanical origin of each fat to avoid sample damage, using in all cases, the minimum temperature and heating time that guaranteed an optimal sample homogenization [19].

### 2.2. Methods

The analysis of M, I, U, acidity, FA composition, triacylglycerols (TAG), diacylglycerols (DAG), monoacylglycerols (MAG), FFA, polymeric compounds (POL), T, and T3 were conducted in duplicate in all AO and FAD samples (Table 2). When available, the official methods for the analysis of crude or refined oils were used, but due to the unusual characteristics of these type of fat samples, they had to be setup and sometimes modified as detailed [19]. The parameters for which no official methods were available were analyzed following standardized protocols available in the scientific literature.

Briefly, M (defined as the % of moisture and any other volatile matter under the conditions of the method) was determined by a vacuum oven method [19,21]. The International Standard ISO 663:2017 was adapted to determine I (g/100 g), in which I stands for the compounds (expressed on wet weight) that are not soluble in petroleum ether 40–60 °C [19,22]. The U content (g/100 g) was determined by saponification and extraction with diethyl ether according to the AOCS official method Ca 6b-53 [23] with some modifications [19]. The weight of the extracted U residue was corrected according to its FFA content (determined by titration with NaOH 0.01 M) expressed as mass of oleic acid. The MIU values (g/100 g) were calculated by summing up M (g/100 g), I (g/100 g), and U (g/100 g) for each sample.

The International Standard ISO 660:2009 was used to measure acidity (FFA-AC), that is to say, FFA content by titration [24]. The FFA-AC was expressed as g of lauric acid/100 g of fat for FAD coming from coconut and palm kernel oils, as g of palmitic acid/100 g for PFAD and as g of oleic acid/100 g for the rest of samples as FA of 18 carbon atoms predominated [19]. To analyze the FA composition, FA methyl esters (FAME) were obtained by a double methylation [25], separated by GC-FID following the conditions described by Tres et al. [29], identified by means of comparison of retention times with those of an external standard mixture (Supelco 37 Component FAME Mix from Merck, Darmstadt, Germany), and quantified by internal area normalization (the quantitative results are obtained by expressing the peak area of a given FA as a percentage of the sum of the areas of all the identified FA peaks). The total saturated (SFA), monounsaturated (MUFA), and polyunsaturated fatty acids (n-6 and n-3 PUFA) were calculated by the sum of the values of individual SFA (C6:0, C8:0, C10:0, C11:0, C12:0, C13:0, C14:0, C15:0, C16:0, C17:0, C18:0, C20:0, C21:0, C22:0, C23:0 and C24:0), *cis*-MUFA (C16:1 n-9, C16:1 n-7, C18:1 n-9, C18:1 n-7 and C20:1 n-9) and *cis*-PUFA (C18:2 n-6, C18:3 n-3, C20:2 n-6, and C22:2). The unsaturated/saturated ratio (U/S ratio) used to predict dietary energy of these fat by-products was calculated according to (*cis*-MUFA + *cis*-PUFA)/SFA. To calculate this ratio, the *trans*-C18:1 isomers (sum of positional isomers) were considered as SFA and as recommended by Wiseman et al. [30] FA with 12 carbons or below were considered as unsaturated FA independently of their degree of saturation.

The TAG (%), DAG (%), MAG (%), FFA-SE (%), and POL (%) in AO and FAD were determined by size molecular exclusion chromatography according to the IUPAC 2508 method [26]. The results of each lipid class were expressed as internal area normalization in % (in relation to the sum of the peak areas of POL, TAG, DAG, MAG, and FFA). The method was applied to all AO, PFAD, and OFAD, but it could not be applied to LFAD samples because the wide range of different molecular weights of TAG, DAG, MAG, and FFA in LFAD meant that their separation by size exclusion columns was not possible [19]. Therefore, in this study, the content of FFA was determined by titration (FFA-AC) as explained above, and by size molecular exclusion chromatography (FFA-SE). 

The amount of T and T3 was determined by HPLC-FLD after saponification [19,27]. Peaks were identified by means of comparison of retention times with those of α-, β-, γ-, and δ-T standards. Quantitation was done through calibration curves built with each T, and the curves were also used also for each corresponding T3. The vitamin E content (expressed as mg of α-T/kg) was calculated by multiplying the individual T and T3 amounts by their respective vitamin E activity conversion factors [31].

Last, the energy of these fat by-products (apparent metabolizable energy, AME, for broilers or the digestible energy, DE, for pigs) was calculated applying the equation suggested by Wiseman et al. [28]:

AME (broilers) or DE (pigs) = A + B (FFA-AC) + C e^D (U/S)^, (1)

where A, B, C, and D are the value of constants for different animal species and ages used in the corresponding prediction equations, with A being a positive coefficient and B, C, D, and E negative coefficients for young and old broilers and pigs; FFA-AC correspond to the FFA contents obtained by titration (acidity), expressed when introduced in this formula as FFA g/kg of fat; and U/S was the ratio unsaturated fatty acids to SFA. As commented above, as recommended by Wiseman et al. [30] to calculate U/S ratio, saturated fatty acids with a carbon chain length equal or shorter than 12 carbons were considered as unsaturated, because they have a similar digestibility. In addition, *trans*-C18:1 isomers were considered as saturated.

Also, energy was calculated by applying another prediction equation that had been based on the Wiseman’s equation [28], but in which the energy was corrected by the MIU (g/100 g) [32]:

(2) $$\mathrm{AME}\;(\mathrm{broilers})\;\mathrm{or}\;\mathrm{DE}\;(\mathrm{pigs})\;=\;(A\;+\;B\;(\mathrm{FFA}-\mathrm{AC})\;+\;C\;e^{D\;(U/S)})\;(1\;-\;\;\frac{\mathrm{MIU}}{100}),$$

### 2.3. Statistics

First, the Shapiro–Wilk test was used to study if the results followed a normal distribution. As data did not follow a normal distribution, non-parametric tests were used for inferential analysis and the mean, standard deviation, median, minimum, and maximum values were considered as descriptive statistical parameters. Mann–Whitney U test was used to compare the distribution of M, I, U, MIU, FFA-AC, FA (SFA, MUFA, n-6 PUFA, n-3 PUFA, and PUFA), n-6/n-3 ratio, U/S ratio, POL, TAG, DAG, MAG, FFA-SE, and T, T3, T + T3, and vitamin E between AO and FAD sample groups.

Kruskal–Wallis test was applied to AO samples and to FAD samples separately to determine if the distribution of the variables was similar between sample groups of different botanical origins and the Stepwise Multiple Comparisons procedure was carried out to compare groups. The distribution of each parameter for AO and for FAD samples of a similar botanical origin was described by using Box-plot graphs. In all cases, *p* <0.05 was considered significant. All univariate data analysis was performed with IBM SPSS Statistics (v 23, IBM, Armonk, NY, USA).

Two Principal Component Analysis (PCA) were conducted on AO and on FAD samples to explore their natural distribution and grouping, to detect outlying samples and to investigate correlations among variables. This method reduces the number of variables to a specific number of principal components (PC), which are linear combinations of the initial variables. Data matrix used in this study consisted of 92 rows (samples) × 32 columns corresponding to the 32 variables for AO: M, I, U, MIU, FFA-AC, main individual FA (C6:0; C8:0; C10:0; C12:0; C14:0; C16:0; C16:1 n-9; C16:1 n-7; C17:0; C18:0; *trans*-C18:1 isomers; C18:1 n-9; C18:1 n-7; C18:2 n-6; C20:0; C18:3 n-3; C20:1 n-9; C22:0; C23:0; C24:0), U/S ratio, POL, TAG, DAG, MAG, FFA-SE, and T + T3 were included. The data matrix used for FAD included 27 variables as POL, TAG, DAG, MAG, and FFA-SE were excluded. All variables were mean centered and scaled to unit variance. The software used for PCA calculation was SIMCA v13.0 (Umetrics AB, Umea, Sweden).

## 3. Results

### 3.1. Variability of the Nutritional Parameters and Differences between Refining Process

Results evidenced a high variability for most of the analyzed parameters. For instance, M, I, and U showed a wide range of values, including samples with very high values both in AO and FAD (Table 3). Accordingly, the global MIU value also showed a high variability, with some FAD samples presenting very low values (0.63 g/100 g) up to some AO samples with MIU values above 18 g/100 g. A high variability was also observed for other parameters such as FA composition, lipid classes, and especially for T contents that in the case of AO ranged from 126.1 to 8464.4 mg/kg (Table 3).

Most parameters significantly differed between AO and FAD, except for I, MUFA, n-6/n-3 ratio, and T3 (Table 3). For instance, M presented higher median and maximum values in AO samples than in FAD. Similarly, AO samples had the highest U median, but the maximum U values were similar between both groups. Consequently, the MIU amount was also higher in AO, although a wide range of MIU values was found in both groups. Also, TAG, DAG, MAG, and POL were higher in samples from chemical refining, and contrarily, FFA-AC and FFA-SE were higher in FAD. Regarding T and T + T3, we observed the highest medians in samples from chemical refining, also when expressed as vitamin E activity.

### 3.2. Sample Clustering According to Botanical Origin

**Figure 1:**
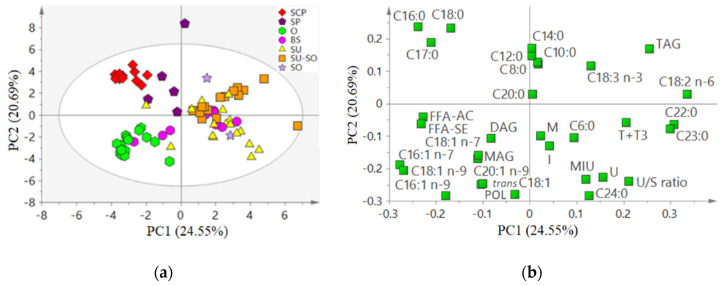
Principal component analysis on the compositional parameters (32 variables, mean centered and scaled to unit variance) of acid oils from chemical refining (AO, *n* = 79). (**a**) Score plot colored according the botanical origin (see Table 1 for abbreviations); (**b**) loading plot (abbreviations: M, moisture; I, insoluble impurities; U, unsaponifiable matter; MIU, sum of M, I, and U; FFA-AC, free fatty acids determined by titration (acidity); POL, polymeric compounds; TAG, triacylglycerols DAG, diacylglycerols; MAG, monoacylglycerols; FFA-SE, free fatty acids determined by size exclusion chromatography; T + T3, sum of α-, β-, γ-, and δ-tocopherols and α-, β-, γ-, and δ-tocotrienols) and U/S ratio, ratio of unsaturated to saturated fatty acids calculated as explained in Table 3.

**Figure 2:**
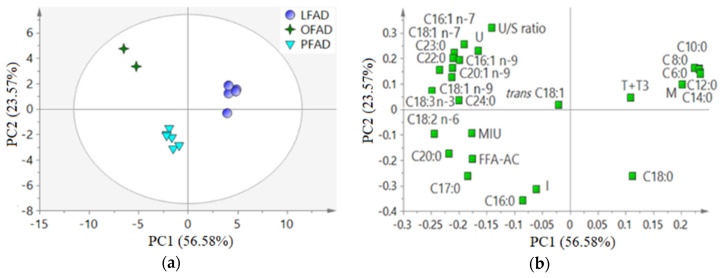
Principal component analysis on the compositional parameters (27 variables mean centered and scaled to unit variance, POL, TAG, DAG, MAG, and FFA-SE were excluded) of fatty acid distillates from physical refining (FAD, *n* = 13). (**a**) Score plot colored according the botanical origin (see Table 1 for abbreviations); (**b**) loading plot (see Figure 1 for abbreviations).

For a global evaluation of the relationships between variables, to study natural clustering of samples and to detect outliers, a PCA was conducted for AO (*n* = 79, Figure 1) and FAD (*n* = 13, Figure 2) samples separately. The cumulative variance explained by the two firsts components of each PCA was 45.24% and 80.15% for the AO and the FAD PCA, respectively.

In the PCA developed with AO samples, SCP, SP, and O formed three groups (being SP more scattered) that were clearly distinguished from the rest (Figure 1a). Both SCP and SP groups agreed with a high contribution of various SFA such as C12:0, C14:0, C16:0, C17:0, or C18:0 (Figure 1b), while the O cluster agreed with a high contribution of various MUFA, mainly palmitoleic (C16:1 n-7) and oleic (C18:1 n-9) acids, together with FFA-SE and FFA-AC (Figure 1b). Samples from the other four botanical groups (BS, SU, SU-SO, and SO) that mainly originated from seed oils, tended to be more scattered and overlapped ones with others (especially BS and SO) (Figure 1a). Their separation agreed with a high contribution of PUFA such as C18:3 n-3 (especially in various SU-SO samples) and C18:2 n-6, C22:0, U/S ratio, and T + T3 (especially in most SU samples). Also, the MIU variable, mainly influenced by U, contributed to the separation of the more unsaturated samples.

In the PCA developed with FAD samples, three sample groups were distinguished and agreed with the three main groups of crude oil’s botanical origin (Figure 2). The two OFAD samples were plotted close to each other because of a high contribution of many unsaturated FA, U/S ratio, and U. Also, PFAD samples formed a cluster in agreement with a high contribution of I and C16:0, among others. The short and medium chain FA (C6:0, C8:0, C10:0, C12:0, C14:0) were correlated and contributed positively to the LFAD clustering, together with M and T + T3.

### 3.3. Differences between Botanical Groups within AO and FAD

Since the PCA results indicated that some clustering agreed with the botanical origin of the corresponding crude oils, we investigated the differences in the compositional and nutritional parameters between botanical groups within the same refining process. Data were visualized by using boxplot graphics.

#### 3.3.1. M, I, U and MIU Values

**Figure 3:**
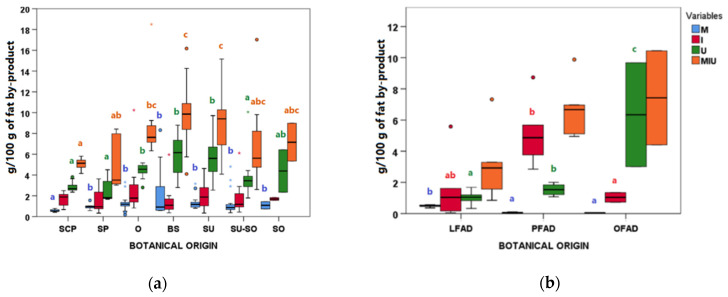
Boxplots for moisture (M), insoluble impurities (I), unsaponifiable matter (U), and the sum of them (MIU) according to botanical groups for (**a**) acid oils from chemical refining (*n* = 79) and (**b**) fatty acid distillates from physical refining (*n* = 13) (see Table 1 for botanical group abbreviations). Within each type of refining and variable, botanical groups bearing different letters (a–c) are significantly different according to Kruskal-Wallis test and post-hoc comparisons (*p* ≤ 0.05).

In AO samples, the lowest M median was observed in the SCP group, which also showed a very low variability (Figure 3a). No significant differences were found between the other AO botanical groups, with the BS the group having the highest M variability, including one outlier with values above 8 g/100 g. Other groups such as O, SU, and SU-SO also showed some extreme outlier samples.

No significant differences were observed for I values among AO botanical groups (Figure 3a), although outliers with I values of 10.24, 6.11, and 5.95 g/100 g were observed in O, SU-SO, and BS groups, respectively.

Regarding U, not only a very high variability was observed within each botanical group, but also significant differences were found between groups (Figure 3a). In this case, SP, SCP, and SU-SO followed by SO were the groups with the lowest U. However, in the SU-SO group, one extreme outlier sample with a U value of 10.06 g/100 g was found. As in general U was the factor that most contributed to the global MIU value, they behaved similarly. Outliers for MIU were found in the O, BS, and SU-SO groups (Figure 3a).

With respect to samples coming from physical refining (Figure 3b), differences between the botanical groups were observed for M, I, and U: The highest M median was found in LFAD, the highest I in PFAD, and the highest U in OFAD, which presented the highest variability. The global MIU showed a similar profile to U, but no significant differences were revealed.

#### 3.3.2. Fatty Acid (FA) Composition

**Figure 4:**
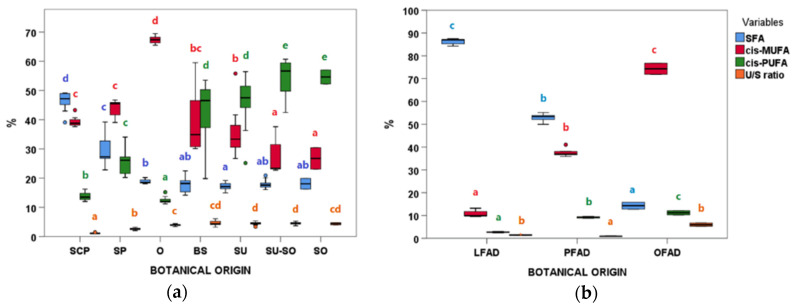
Saturated fatty acids (SFA), monounsaturated fatty acids (MUFA), polyunsaturated fatty acids (PUFA), and unsaturated/saturated ratio (U/S ratio) boxplots according to botanical groups for (**a**) acid oils from chemical refining (*n* = 79). and (**b**) fatty acid distillates from physical refining (*n* = 13) (see Table 1 for botanical group abbreviations). Within each type of refining and variable, botanical groups bearing different letters (a–e) are significantly different according to Kruskal–Wallis test and post-hoc comparisons (*p* ≤ 0.05).

The effect of the botanical origin on the FA profile is shown in Figure 4. For SFA, differences were observed between botanical groups within AO samples, with SCP and SP being the groups with higher SFA (%) amounts (Figure 4a). The variability within each group was different, with SP being the one showing the widest range of SFA percentages. In FAD, SFA showed a narrower variability range and differences were also observed within groups (Figure 4b), with LFAD and PFAD being the richest in SFA.

Regarding MUFA, samples from olive and olive pomace oil refining (O and OFAD) reached the highest values in both refining processes, followed by samples containing palm oil in their composition (SP, SCP, PFAD) (Figure 4). It was remarkable the wide MUFA ranges observed for the BS group, followed by SU-SO whose median values were very different (Figure 4a).

Regarding PUFA, AO samples (Figure 4a) coming mainly from seed oils (SU-SO, SO, SU, and BS) had higher and more variable values than the other groups. In FAD (Figure 4b), OFAD presented the highest PUFA median. Differences were observed for the n-6/n-3 ratio being higher and highly variable in BS, SU, SU-SO, and SO (Figure S1, Supplementary Material). The *trans*-C18:1 isomers were higher in AO than in FAD (Table 3). The lowest U/S ratios were found for SCP and SP, with BS, SP, and OFAD being the ones with the highest variability (Figure 4).

#### 3.3.3. Lipid Classes (FFA-SE, MAG, DAG, TAG, and POL) and Acidity (FFA-AC)

**Figure 5:**
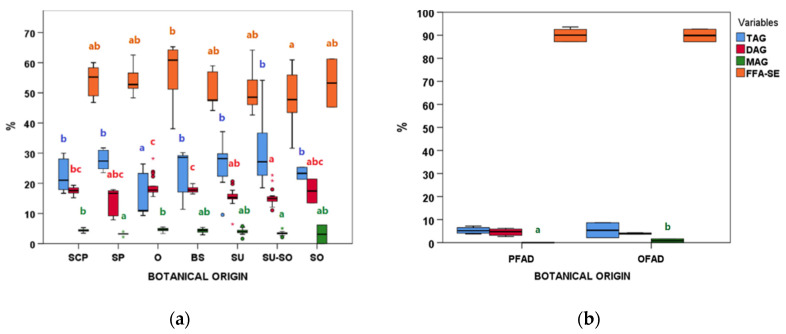
Triacylglycerols (TAG), diacylglycerols (DAG), monoacylglycerols (MAG), and free fatty acids (FFA-SE) boxplots according to botanical groups for (**a**) acid oils from chemical refining (*n* = 79) and (**b**) fatty acid distillates from physical refining (*n* = 8, lauric FAD group was excluded) (see Table 1 for botanical group abbreviations). Within each type of refining and variable, botanical groups bearing different letters (a–c) are significantly different according to Kruskal–Wallis test and post-hoc comparisons (*p* ≤ 0.05).

In all cases, FFA-SE was the richest lipid fraction, followed by TAG, DAG, and MAG (Figure 5). They varied in wide ranges even within the same AO botanical group (Figure 5a). Even if the ranges of different AO groups were overlapped, some significant differences in their distributions were observed (Figure 5a). Regarding FFA-AC, it behaved similarly to FFA-SE (Figure S3, Supplementary Materials), as the main contributor to FFA-AC values were FFA. For samples from physical refining, significant differences were found comparing MAG medians between PFAD and OFAD (Figure 5b). The lowest FFA-AC median within FAD was found in the LFAD group, which showed a higher variability that the other FAD groups.

Regarding POL, all AO groups presented relatively low medians (in all cases, below 4%), but again, a high variability (Figure S2, Supplementary Material). In many of them, samples with null values and also samples with relatively high values, even above 6% in O and SU, were found. As commented above, no POL were detected in FAD samples.

#### 3.3.4. Tocopherol (T) and Tocotrienol (T3) Content

**Figure 6:**
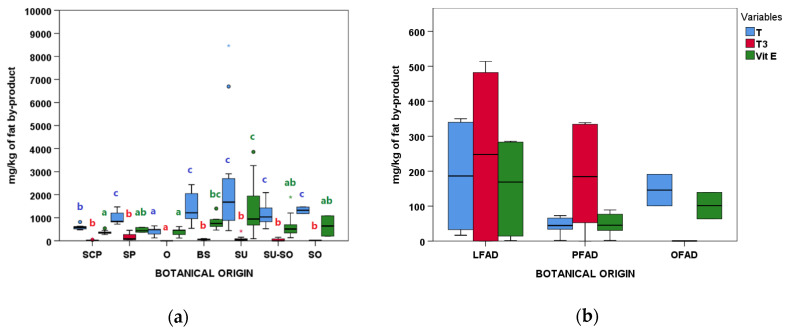
Tocopherols (T), tocotrienols (T3), and Vitamin E content according to botanical groups for (**a**) acid oils from chemical refining (*n* = 79) and (**b**) fatty acid distillates from physical refining (*n* = 13) (see Table 1 for botanical group abbreviations). Within each type of refining and variable, botanical groups bearing different letters (a–c) are significantly different according to Kruskal–Wallis test and post-hoc comparisons (*p* ≤ 0.05).

Among AO groups, O and SCP had lower T contents than the rest, while no differences were observed within FAD (Figure 6a). Differences were also found for the individual T and T3 (Table S1, Supplementary Material). For instance, O had the lowest γ-T and no α-T3 was found in O or OFAD groups. It was surprising that two SU samples presented very extreme T values of 6694.9 and 8464.4 mg/kg, especially compared to the other AO in the SU group and to sunflower oil [33]. The high variability in T contents was remarkable, especially for SU, BS, and SU-SO groups in AO, and in T and T3 contents in LFAD.

Overall, the vitamin E activity that was mainly determined by the α-T content (Table S1, Supplementary Material) was higher in the SU group followed by BS, SU-SO, SO, and SP groups (Figure 6). Similar to T results, vitamin E activity did not show significant differences between FAD groups. Again, the high variability observed for some groups such as SU, BS, or LFAD was remarkable.

#### 3.3.5. Dietary energy of These Fat by-products

**Figure 7:**
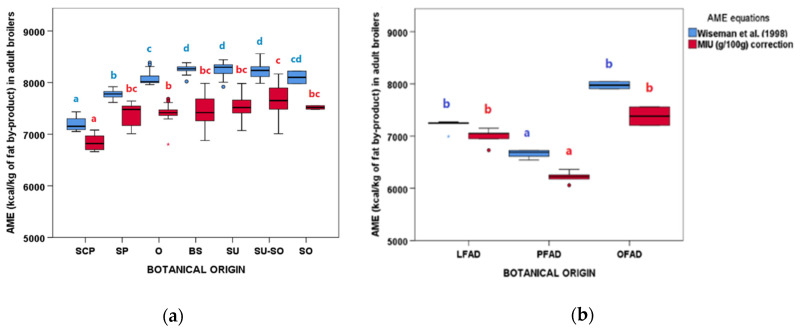
Apparent metabolizable energy (AME) calculated for adult broilers according to Wiseman’s equation [28] and by applying the MIU (g/100 g) correction to it [32] of (**a**) acid oils from chemical refining (*n* = 79) and (**b**) fatty acid distillates from physical refining (*n* = 13). See Table 1 for botanical group abbreviations. Within each type of refining and variable, botanical groups bearing different letters (a–d) are significantly different according to Kruskal–Wallis test and post-hoc comparisons (*p* ≤ 0.05).

The AME for broilers and DE for pigs of the fat by-products were calculated applying Wiseman’s equation [28] and correcting it by the MIU content [32] (Figure 7 and Table S2, Supplementary Material). Differences were observed between groups, both for AO and for FAD. In AO, SCP samples showed the lowest calculated energy, followed by SP. In FAD, the lowest calculated energy was found for PFAD samples, followed by LFAD and OFAD. In all cases, these values decreased when the MIU correction was applied. It was also remarkable that after MIU correction, the ME and DE variability increased (Figure 7 and Table S2, Supplementary Material).

## 4. Discussion

Results have shown that some of the compositional parameters assessed in AO and FAD, such as FFA-AC, the lipid classes (TAG, DAG, MAG, and FFA-SE) or M, mainly varied depending on the refining process, while others such as T, T3, U, and FA composition, were more influenced by the botanical source of the oil originating these by-products.

### 4.1. Influence of the Refining Process

Both AO and FAD are the by-products from the steps of chemical and physical refining in which FFA are removed from crude oil, and thus both were rich in FFA, leading to high FFA-AC and FFA-SE. However, while in chemical refining (AO), FFA are mainly removed by neutralization and centrifugation and usually not exceeding 100 °C, in physical refining, they are removed during deodorization by distillation, applying vacuum and temperatures around 180–270 °C [1,2,3,34]. Thus, these different FFA removal processes might have also influenced AO and FAD composition, especially the FFA-AC and the FFA-SE contents that were much higher in FAD (78–94% for FFA-SE) than in AO (31–65% for FFA-SE), in agreement with Nuchi et al. [4]. On the contrary, TAG, DAG, MAG, POL, and M contents were higher in AO. Since FAD are originated from a distillation process, they might accumulate compounds such as FFA, as well as secondary oxidation compounds, T and T3, hydrocarbons, or sterols, that can distillate at the process conditions [3]. On the contrary, during the separation of soap-stocks from the refined oil by centrifugation in the neutralization step of chemical refining, a similar amount of neutral oil (50%) might be concomitantly washed away together with FFA, M, phospholipids, U compounds, proteins, and other mucilaginous substances [6,35]. However, as it will be discussed below, the botanical origin of the oil might also influence some of these parameters such as T and T3. On the other hand, the higher temperatures reached during deodorization in physical refining might have favored that water was less retained in FAD leading to median values of 0.97 g/100 g and 0.07 g/100 g for AO and FAD groups, respectively, and reaching values as high as 8.32 g/100 g in some outlying AO samples. The M values of AO could also be influenced by the presence of phospholipids. The presence of phospholipids in some AO samples could not be excluded as it is known that hydrated phospholipids from degumming step might be added to AO [36]. Thus, they could be contributing to higher water content in the AO.

### 4.2. Influence of Crude Oil Botanical Origin

As it has been explained above, process conditions in physical refining are usually more drastic than in chemical refining, especially when referring to process temperature that might favor lipid oxidation [1,3]. Consequently, chemical refining is usually preferred for unsaturated oils such as soybean, sunflower, or rapeseed, which in turn are also naturally richer in T than saturated fats [33], while physical refining is commonly used for less unsaturated fats such as palm or lauric oils [3,37]. Thus, although the processing conditions between chemical and physical refining differ, the different FA and T + T3 composition of crude oils (botanical origin) might have a major role in the FA composition and in T and T3 contents of AO and FAD. For instance, regarding tocols, it is known that, during refining, the tocol removal mainly occurs in the deodorization step followed by the neutralization step [1,38,39]. Thus, it would have been logical to expect higher tocols in FAD than in AO; but since AO were mainly obtained from seed oils and FAD from palm and lauric oils, AO were richer in tocols. Remarkably, tocols were higher in AO also when expressed as vitamin E, being from 2 to 20 times higher than in FAD. This, together with the higher POL in AO, contributed to the higher U contents in samples from chemical refining, and to the fact that U and tocols were correlated in the PCA.

The influence of the botanical origin of the crude oil also explained the higher SFA and lower *cis-*PUFA globally observed in FAD. Indeed, the influence of the crude oil botanical source was evident in the tocol and FA composition within the various AO and FAD groups. The highest *cis-*PUFA corresponded to AO coming from the most unsaturated sources, such as soybean oils (SU-SO and SO) followed by other seed oils (SU and BS). All of them were richer in C18:2 n-6 and C18:3 n-3 than the other AO groups as these FA are present in high amounts in sunflower and soybean oils. The same trend was observed for T, in agreement with the high T contents in seed oils. Accordingly, SU, followed by BS, were the richest in vitamin E as they were also the richest in α-T, which is the tocol with the highest vitamin E activity. On the other hand, the highest *cis-*MUFA contents were observed in the olive oil by-products (compared to other AO and FAD), and also the separation of O and OFAD clusters in their respective PCAs was driven by C18:1 n-9, C16:1 n-9, and C20:1 n-9 that are FA characteristic of olive oil. Then, the second highest *cis-*MUFA in both refining processes corresponded to those groups that contained palm oil by-products (SCP, SP, PFAD) because although palm oil is frequently categorized as a saturated fat, it contains equal proportions of saturated (mainly palmitic acid) and unsaturated acids (mainly oleic acid) [33]. Differences between SFA were also related to the oil source: SCP group was the richest AO in total SFA and in palmitic and stearic acids as they are typical of cocoa butter while LFAD group was the richest FAD in SFA as a result of its high content in medium chain SFA reflecting the FA composition of coconut and palm-kernel oils [33].

The *trans*-C18:1 isomers were significantly higher in AO than in FAD (Table 3), which was unexpected as the formation of *trans* isomers during oil refining is mainly due to the high temperatures applied during deodorization. However, on the one hand, it needs to be taken into account that FAD showed lower *trans*-C18:1 isomers because they include LFAD samples (Table 1), which are poor in C18:1 n-9 and C18:1 n-7. On the other hand, seven out of the eight AO samples with a *trans*-C18:1 isomer content higher than 2% were AO from the refining of olive pomace oils. Olive pomace is usually obtained from the two-phase centrifugation method used in olive oil extraction (by large the method of olive oil extraction more used in Spain). It is a very wet pomace (called in Spain *alperujo*) that might contain up to 60 ± 5 g of moisture/100 g, and it must be dried down to 7–8 g of moisture/100 g to reach an optimum extraction yield of olive pomace oil. Usually, this drying is carried out using rotary dryers that consist of a large cylinder where hot and dry air enters at a high temperature (450 ± 50 °C) and leaves humid at 95 ± 5 °C [40,41], drying conditions that might have contributed to the formation of an important part of these *trans*-C18:1 isomers. The amount of *trans* FA in feed fats is of relevance as it might influence the final *trans* FA in meat of monogastric animals [42]. The current regulation in the EU has set a maximum limit in foods of 2 g (of *trans* FA, other than *trans* FA naturally occurring in fat of animal origin) per 100 g of fat [43]. Studies conducted in chickens fed feeds containing PFAD with a 0.5 or 5.2% of *trans* FA, which are values similar to the maximum values found for FAD and AO in this study, led to thigh meat (with skin) with 0.3 and 1.3 g of *trans* FA/ 100 g of fat, respectively [42], which are values below the current limit.

The U/S ratio showed similar results to PUFA in AO. The U/S ratio is a relevant parameter in the field of animal feeding because, generally, when U/S ratio increases, the fat digestibility increases as well [44,45]. Unsaturated FA not only have greater digestibility and absorption rates than SFA, but it is also believed that they might improve SFA digestibility by increasing the micelle formation when SFA are present in the diet [17,46]. However, the effect is not linear as it depends on the animal’s age and species [46]. Indeed, it has been reported a better utilization of unsaturated FA than SFA in broiler chickens, as well as a better ability to digest and absorb fat when the animal age increases [14,17]. In the case of lauric oils, even if they have a high SFA content, they are highly digestible because of their high content in short chain FA [47]. This is why Wiseman et al. [30] recommended to include SFA with 12 carbon atoms or less as unsaturated FA in the U/S ratio for the energy calculation, and thus, this explained that within our FAD, the U/S ratio was lower for PFAD than for LFAD. Thus, considering AO and FAD results, it would be advisable to blend the more saturated products (part of SP samples, SCP, and PFAD) with a more unsaturated seed oil to increase the U/S ratio and improve digestibility. The optimal proportion of the blend needs to be set depending on the animal species, age, and blended products, and it also needs to be determined if this blend is more appropriate to be done with an oil or with an oil by-product (such as OFAD, O, SU, SU-SO, SO or BS). However, it needs to be taken into account that high U/S ratios especially as a result of the presence of high PUFA contents, may lead to oxidative damage, predominantly in younger birds, and might affect broiler performance and meat quality [48]. However, on the other hand, some PUFA such as linoleic and linolenic acids are considered metabolically essential because birds are not able to synthesize them.

The presence of palm or corn oil by-products (especially in SP but also in SCP) also agreed with high T3 values, particularly γ-T3, which is a tocol analogue characteristic of palm oil. It was remarkable that T and T3 in LFAD reached even higher contents than those found in our PFAD or in coconut and palm kernel oils [33]. However, on the other hand, even if LFAD was the richest FAD in tocols and OFAD the lowest, in PCA, U was more correlated with OFAD. This would indicate that other U compounds such as squalene might have accumulated in OFAD during distillation. According to Psomiadou and Tsimidou [49] squalene is the major olive oil hydrocarbon, which could be present in more than 50% of the U content of the crude olive oil.

It is important to highlight the high variability observed for the FA composition, T + T3 contents and U, especially for the SU, BS, and LFAD groups. Various samples in the SU group had C18:3 n-3, γ-T, and T3 contents higher than those expected for a sunflower oil by-product, suggesting a presence of soybean by-products in some SU samples. On the other hand, the extremely high tocol contents of the two SU outliers could agree with the addition of tocol-rich products, such as deodistillates coming from the deodorization step in chemical refining [39]. However, deodistillates from chemical refining are not included among the products listed in the EU Catalogue of feed materials [7]. Regarding the variability observed for the LFAD group, it has to be taken into account that LFAD included two types of samples: Two pure coconut FADs of which T + T3 contents were below 100 mg/kg in agreement with the very low T3 amounts reported for coconut oil; and three blends of coconut and palm kernel FADs whose T + T3 contents widely differed (reaching up to 850 mg/kg) even if they had been provided by the same company. Unfortunately, for many AO and FAD blends (such as BS, LFAD, SCP, or SP), the exact proportion of each oil source in the blends declared by the producers was approximate or even unknown. In refineries that refine various types of oils, the same waste tank may collect soap-stocks from different oils that are later acidified and transformed into AO, and the same might happen with tanks collecting FADs. In addition, the homogeneity in these tanks is poor, especially in the case of soap-stocks, which are very viscous and form layers corresponding to the different refining batches. In other cases, fat producers may intentionally mix various AO to achieve a certain final composition for the blend. Hence, the natural variability of crude oils and the influence of process and storage conditions could lead to highly variable tocol amounts, and thus, it would be a challenge for AO and FAD producers to standardize the content of tocols and other nutrients. Nevertheless, the tocol content is an important point in animal feeding, because of its antioxidant properties and vitamin E activity [45]. Thus, this suggests that feed producers should be aware of this high variability, demanding this information to the producer so that when the expected amounts are not reached, they can be corrected by supplementing feeds with tocopherols.

### 4.3. Comparison with Fat Quality Thresholds in EU Regulations and Guidelines

Samples included in this study had been collected from the Spanish market and were expected to be compliant with the EU Catalogue of feed materials [7] and to follow the FEDNA guidelines [12]. The FEDNA guidelines state a maximum of 0.15 g/100 g for I measured by the ISO663:2017 method for various AO and FAD [12,22]. Even if in this study I was measured by this method, only one sample out of the 92 collected had an I content below this threshold. The high variability in I contents even between samples from similar botanical origins and refining process was remarkable. The presence of I in AO and FAD could be related to the I contents of crude oil, to processing contamination, or to fragments of some gums such as lecithins that some refineries might add to AO to dispose them and to seek an improvement of fat utilization by the animal through their emulsifying properties [9,36]. Therefore, it is evident that this type of sample tends to accumulate high amounts of these I compounds as they are refining by-products. Considering that the median for I was 1.65 g/100 g, and the 90th percentile was 3.80 g/100 g, it would be advisable to revise the thresholds stated in the guidelines for this type of by-products.

Regarding M, it was above 1 g/100 g in 38 samples and thus, according to the EU Catalogue of feed materials, they were M amounts compulsory to declare [7]. The U or MIU contents are currently not specifically included in the EU regulation of feed fats [7], but the FEDNA guidelines recommend MIU values lower than 5 g/100 g both for AO and FAD [12]. However, only 23 samples agreed with this recommendation (17 AO and 6 FAD samples). Overall, only one sample was below the limits for M, I, or MIU in the EU Catalogue of feed materials or in the FEDNA guidelines [7,12]. Other parameters such as the FFA-AC content and the FA profile, including the U/S ratio, the n-6/n-3 ratio, and FA chain length, have also been described as nutritionally relevant for animal feeding as they might influence fat digestibility and, thus, energy utilization [46]. However, none of these parameters are specified in the EU Catalogue of feed materials [7].

### 4.4. Prediction of Dietary Energy Value of These Fat by-Products

The energy value of an animal diet can be calculated by the energy value of each ingredient reported in feeding tables from official organisms or be estimated through equations that relate the energy value to the chemical characteristics of the ingredients. Wiseman et al. [28] developed the most relevant and useful prediction equation so far for two species (broilers and pigs) and for two ages (young and old). The equation predicts AME (for broilers) and DE (for pigs) based on U/S ratio and fat FFA-AC content, derived through curvilinear regression analysis. As explained above, the U/S ratio is relevant for the energy value of a fat and it depends on its botanical origin. Thus, this caused the energy of AO and FAD to be higher in the more unsaturated groups (SU, SU-SO, SO, BS, and O and OFAD). The lowest calculated energy values were found for PFAD, followed by LFAD that were rich in SFA of 12 C atoms or less [47]. As these FA are highly digestible, they were considered as unsaturated in the U/S ratio as reported by Wiseman et al. [30] in order to avoid underestimating the energy of lauric oils as no other equations have been described for this type of oils so far.

The estimation of the energy value also considers the FFA-AC content, which is one of the most distinct compositional traits between these by-products. Various studies relate FFA with impairments of energy value, fat digestion, and absorption. For instance, back into 1979, Sklan et al. [50] reported that feeding oils rich in FFA led to both a reduction of MAG contents in the intestinal lumen and also of endogenous bile secretion, which resulted in a poorer energy utilization because MAG is needed to solubilize and absorb FFA. Later, Wiseman, and Salvador [13] established that FFA progressively reduced the AME of AO and FAD, and that also the U/S ratio influenced it, especially in young birds. Later, Vilà and Esteve-Garcia [51] found no significant differences in feed efficiency between the use of oils rich in FFA and refined oils, and recently, Rodriguez-Sanchez et al. [14,17] found that SFA had a greater impact on FA absorption than FFA, and that the FFA utilization improved with the age of the animal.

Apart from this, also the dilution role of oxidized FA and POL on AME has been recognized, and these and some other U and NEM compounds have been negatively correlated with AME [52,53]. Therefore, it can be concluded that U/S ratio and FFA alone would not be a good measure of ME in broilers and that increases in energy diluent compounds might lead to a reduction of the dietary energy [51,52]. Thus, modifications of the Wiseman’s equation have been suggested, for instance to correct the energy by the MIU content [32]. When the energy of AO and FAD was corrected for their MIU contents, the energy of the most unsaturated groups (BS, SU, SU-SO, SO, and O) decreased to values similar to those of SP group, all still being higher than that of SCP. In FAD, the decrease was more pronounced in OFAD and PFAD, with PFAD still being the one with the lowest energy values. Thus, the decrease depended on the oil origin and refining process, and it could be as high as 806 kcal/kg in MIU rich samples. This highlights that a proper energy calculation by equations should include the values of the energy diluents, especially when working with MIU rich by-products such as AO and FAD, so that feed formulations can be adjusted accordingly.

It also needs to be taken into account that the content of most of these components in these by-products is sometimes erratic even between batches from the same company as they depend on the natural variability of the raw materials and on the refining process conditions. Even if the refining process is optimized to obtain a standardized refined oil, any effort towards the standardization of these by-products could contribute to increase the confidence of animal nutritionists in them. This standardization could imply the optimization of the by-product processing, or the correction of the contents of some nutritional relevant compounds such as essential FA or tocols.

## 5. Conclusions

The use of fat by-products from edible oil refining, such as AO and FAD, as feed fats might be a way to upcycle and valorize these by-products, and to increase feed energy and its content of liposoluble vitamins and other essential lipid nutrients. However, the different way of removing FFA by chemical or physical refining also caused other compounds to be concomitantly washed away (or not) from the refined oil, thus affecting the global composition of AO and FAD. Overall, our results reflect a relationship between the main components of these by-products and the production technology. As FAD originated from a distillation step, they showed higher FFA-AC and FFA-SE, whereas AO maintain higher proportions of POL, TAG, DAG, and MAG from the initial oil as they are concomitantly washed away with FFA during alkali neutralization. Thus, depending on the obtention process, the ratio between FFA and the rest of the lipid fractions might vary considerably. However, the content of other compounds with nutritional relevance, such as FA and tocols, was more dependent on the botanical origin of the corresponding crude oils. More interestingly, for most of these compounds there was a great variability in their values, even between batches from the same producer, which might make it difficult for animal nutritionists to get a standardized product that they can easily include in feed formulations. Furthermore, it would be advisable to revise the limit stated for I in the guidelines for these by-products, as almost all samples were above it. In many samples (69 out of 92), MIU values were also above those recommended by FEDNA guidelines (5 g/100 g) [12], and MIU values as high as 18 g/100 g could be reached. Thus, it would be advisable to estimate the energy of fats considering the diluent effect of MIU compounds, because otherwise the calculation might be overestimated even by 806 kcal/kg depending on the type of refining process and the botanical origin of the oil. Therefore, it is necessary to standardize these fat by-products to increase animal nutritionists’ confidence in them, and the industry should be encouraged to optimize their production practices to achieve products with less variable composition.

## Figures and Tables

**Table 1:** Sample’s classification according to the refining process and botanical origin.

Refining Process	Group	Botanical Origin	Subgroup: Different Mixtures	*N*	Total	
Chemical refining (Acid oils, AO)	SCP	Blends of AO from seed oils, cocoa butter, and palm oil ^1^	Cocoa butter, rapeseed, soybean, and palm oils (40/30/20/10)	2	12	
			Cocoa butter, palm and seed oils	10		
	SP	Blends of AO from seed and palm oils ^1^	Soybean, rapeseed, and palm oils (40/40/20)	2	5	
			Sunflower, soybean, palm, corn, and rapeseed oils	3		
	O	AO from olive pomace oil and blends of AO from olive pomace and olive oils	Olive pomace oil	13	18	
			Olive pomace and olive oils (90/10)	5		
	BS	Blends of AO from seed oils ^1,2^	Sunflower (80–90), rapeseed (20–10) and traces of palm and palm kernel oils and palm stearin	1	9	
			Sunflower, corn, and grapeseed oils (40/30/30)	3		
			Sunflower, soybean, and corn oils	3		
			Sunflower, high oleic sunflower, soybean, corn, and olive pomace oils	2		
	SU	AO from sunflower oil	Sunflower oil	18	18	
	SU-SO	Blends of AO from sunflower and soybean oils ^1^	Sunflower and soybean oils	4	15	
			Sunflower and soybean oils (10/90)	7		
			Sunflower and soybean oils (80/20)	2		
			Sunflower and soybean oils (90/10)	2		
	SO	AO from soybean oil	Soybean oil	2	2	
Physical refining (Fatty acid distillates, FAD)	LFAD	FAD from coconut oil and blends of FAD from coconut and palm kernel oils ^1^	Coconut oil	2	5	
			Coconut and palm kernel oils	3		
	PFAD	FAD from palm oil	Palm oil	6	6	
	OFAD	FAD from olive pomace and olive oils	Olive pomace oil	1	2	
			Olive oil	1		

^1^ For some blends the proportions were unknown; ^2^ Some blends contained traces of fruit oils. Abbreviations: see the “Botanical origin” column for the definition of abbreviations.

**Table 2:** Analytical methods used to determine the quality parameters of acid oils and fatty acid distillates.

Analytical Method	Main Reference ^1^
Sample preparation	Varona et al., under review [19]
M	AOCS Official Method Ca 2d-25 [21]
I	ISO 663:2017 [22]
U	AOCS Official Method Ca 6b-53 [23]
FFA-AC	ISO 660:2009 [24]
FA composition	Guardiola et al. [25]
POL, TAG, DAG, MAG and FFA-SE	IUPAC, standard method 2508 [26]
T and T3	Aleman et al. [27]
Dietary energy, calculated	Wiseman et al. [28]

^1^ All protocols are detailed in [19]. Abbreviations: M, moisture and volatile matter; I, insoluble impurities; U, unsaponifiable matter; FFA-AC, acidity; FA, fatty acid; POL, polymeric compounds; TAG, triacylglycerols; DAG, diacylglcyerols; MAG, monoacylglycerols; FFA-SE, free fatty acids fraction; T, tocopherols; T3, tocotrienols. Note: the content of FFA was determined by titration (FFA-AC) and by size molecular exclusion chromatography (FFA-SE; IUPAC, standard method 2508)

**Table 3:** Mean, standard deviation, median, minimum, and maximum values obtained for acid oils (AO) and fatty acid distillates (FAD).

Parameter	Acid Oils (AO, *n* = 79)				Fatty Acid Distillates (FAD, *n* = 13)				*p* ^1^
	Mean ± SD	Median	Min	Max	Mean ± SD	Median	Min	Max	
M (g/100 g)	1.31 ± 1.25	0.97	0.17	8.32	0.12 ± 0.12	0.07	0.00	0.37	0.000
I (g/100 g)	1.95 ± 1.51	1.57	0.33	10.24	3.17 ± 2.65	2.85	0.05	8.74	0.228
U (g/100 g)	4.36 ± 1.89	4.20	1.67	10.06	2.07 ± 2.38	1.34	0.32	9.67	0.000
MIU (g/100 g)	7.62 ± 3.22	7.35	2.60	18.50	5.37 ± 3.00	5.11	0.63	10.44	0.032
FFA-AC (g/100 g) ^2^	57.4 ± 8.06	57.0	36.4	74.7	79.7 ± 9.58	82.5	64.5	92.2	0.000
SFA (%) ^3^	23.0 ± 10.62	18.5	14.2	49.1	59.8 ± 25.74	53.8	12.8	87.6	0.000
*cis*-MUFA (%) ^3^	43.4 ± 15.72	38.9	22.8	70.7	33.2 ± 22.43	36.9	9.8	76.9	0.098
n-6 PUFA (%) ^3^	32.2 ± 18.05	36.8	10.2	59.2	6.7 ± 3.39	8.5	2.3	10.9	0.000
n-3 PUFA (%) ^3^	1.4 ± 1.03	1.1	0.2	5.3	0.3 ± 0.32	0.3	0.1	1.2	0.000
n-6/n-3 ratio	33.5 ± 43.15	18.5	9.3	312.7	27.6 ± 10.80	26.0	8.9	54.3	0.246
*cis*-PUFA (%) ^3^	33.6 ± 18.52	37.3	11.2	60.7	7.0 ± 3.64	8.9	2.4	12.1	0.000
*trans*-C18:1 (%)	0.9 ± 0.82	0.66	N.D.	4.5	0.2 ± 0.10	0.2	0.1	0.5	0.000
U/S ratio ^4^	4.0 ± 1.39	4.4	1.0	6.2	1.9 ± 1.90	1.4	0.8	6.8	0.001
POL (%) ^5^	2.6 ± 1.58	2.5	ND	6.8	ND	ND	ND	ND	0.000
TAG (%) ^5^	23.8 ± 8.61	24.6	9.3	54.2	5.3 ± 2.12	5.2	2.1	8.6	0.000
DAG (%) ^5^	16.8 ± 3.25	16.9	6.5	28.2	4.4 ± 1.34	4.0	2.6	6.3	0.000
MAG (%) ^5^	4.1 ± 0.98	4.2	ND	6.2	0.2 ± 0.57	0.0	ND	1.6	0.000
FFA-SE (%) ^5^	52.7 ± 7.73	52.9	31.7	65.3	90.1 ± 2.85	90.0	87.2	93.6	0.000
T (mg/kg)	1167.1 ± 1234.52	813.1	126.1	8464.4	113.8 ± 117.93	65.6	1.8	350.1	0.000
T3 (mg/kg)	48.3 ± 80.21	21.8	ND	454.7	179.9 ± 194.13	89.3	ND	514.0	0.065
T+T3 (mg/kg)	1215.4 ± 1247.57	840.7	126.1	8514.1	293.7 ± 287.64	192.0	1.8	853.7	0.000
Vitamin E (mg/kg) ^6^	707.8 ± 659.12	489.5	86.7	3855.9	95.7 ± 97.32	63.6	1.1	285.6	0.000

Abbreviations: M, moisture and volatile matter; I, insoluble impurities; U, unsaponifiable matter; MIU, sum of moisture, insoluble impurities and unsaponifiable matter; FFA-AC, free fatty acids determined by titration (acidity); SFA, saturated fatty acids; MUFA, monounsaturated fatty acids; PUFA, polyunsaturated fatty acids; n-6/n-3 ratio, n-6 polyunsaturated/n-3 polyunsaturated; U/S ratio, unsaturated/saturated ratio; POL, polymeric compounds; TAG, triacylglycerols; DAG, diacylglycerols; MAG, monoacylglycerols; FFA-SE, free fatty acids determined by size exclusion chromatography; T, sum of α-, β-, γ- and δ-tocopherols; T3, sum of α-, β-, γ- and δ-tocotrienols; T + T3, sum of tocopherols and tocotrienols; ND, not detected; ^1^ *p* values were obtained from Mann Whitney U test for independent samples to compare medians between both refining groups. *p* ≤ 0.05 was considered significant; ^2^ FFA-AC (acidity) was expressed as g of oleic acid/100 g in all samples except for lauric FAD (g of lauric acid/100 g) and PFAD (g of palmitic acid/100 g); ^3^ Table shows the sums of fatty acids including all the identified and quantified FA, expressed as internal area normalization in %: C6:0, C8:0, C10:0, C11:0, C12:0, C13:0, C14:0, C15:0, C16:0, C16:1 n-9, C16:1 n-7, C17:0, C18:0, *trans*-C18:1 (sum of positional isomers), C18:1 n-9, C18:1 n-7, C18:2 n-6, C20:0, C18:3 n-3, C20:1 n-9, C21:0, C20:2 n-6, C22:0, C23:0, C22:2, C24:0; ^4^ To calculate the U/S ratio to predict the dietary energy of these fat by-products through Wiseman’s equation [28] the *trans*-C18:1 isomers were considered saturated and as recommended by Wiseman et al. [30] FA with 12 carbons or below were considered as unsaturated FA independently of their degree of saturation. ^5^ In the case of the variables corresponding to lipid classes (POL, TAG, DAG, MAG and FFA-SE) of FAD, *n* = 8 because these variables could not be determined in the lauric FAD samples (*n* = 5). In all cases, lipid fractions were expressed as internal area normalization in %; ^6^ The total vitamin E activity (expressed as mg of α-tocopherol/kg) was calculated using the activity conversion factors given by McLaughlin and Weihrauch [31] for each T and T3.

## Data Availability

Not applicable.

## Supplementary Materials

The following are available online at https://www.mdpi.com/2076-2615/11/1/196/s1, Figure S1: n-6 Polyunsaturated/n-3 polyunsaturated fatty acid ratio (n-6/n-3 ratio) boxplots according to botanical groups; Figure S2: Polymeric compounds (POL) boxplots according to botanical groups; Figure S3: Acidity (FFA-AC) boxplots according to botanical groups; Table S1: Median values for the individual tocopherols, tocotrienols and vitamin E content (mg/kg) according to the refining process and botanical origin; Table S2: Median values of apparent metabolizable energy (AME, kcal/kg) for broilers and digestible energy (DE, kcal/kg) for pigs of different ages (young and adult) of acid oils and fatty acid distillates. AME and DE values were obtained according to Wiseman et al. [28] equation and by applying the MIU (g/100 g) correction to it as suggested by Bierinckx [32].
