# Supplementary material for: Composition and Nutritional Value of Acid Oils and Fatty Acid Distillates Used in Animal Feeding
Source: Animals (Basel). 2021 Jan 15;11(1):196. doi: 10.3390/ani11010196 (PMC7830271; doi:10.3390/ani11010196)
Supplement: Supplementary file 1 [file animals-11-00196-s001.pdf]

Supplementary Material for the manuscript:

Varona et al., Composition and nutritional value of acid oils and fatty acid distillates used in animal feeding,

submitted to *Animals*

Table S1. Median values for the individual tocopherols, tocotrienols, and vitamin E content (mg/kg) according to the refining process and botanical origin.

Botanical origin		α-T	β-TF	ϒ-TF	δ-TF	α-T3	β-T3	ϒ-T3	δ-T3	T	T3	T+ T3	Vitamin E[1]
Chemical refining (AO, n=79)	SCP	320.9[a]	11.2[b]	222.3[b]	20.7[b]	7.6[b]	ND	15.9[b]	ND	576.3[b]	23.9[b]	598.6[b]	353.6[a]
	SP	340.3[ab]	26.4[c]	237.7[b]	249.3[c]	21.5[b]	ND	43.9[b]	17.8[b]	839.1[c]	83.2[b]	1081.2[c]	434.7[ab]
	O	449.3[ab]	9.1[a]	17.2 [a]	ND	ND	ND	ND	ND	477.1[a]	ND	477.1[a]	455.0[a]
	BS	591.9[bc]	31.0[c]	236.6[b]	28.3[bc]	29.6[b]	ND	26.2[b]	ND	1211.4[c]	55.8[b]	1239.7[c]	753.2[bc]
	SU	883.9[c]	51.5[c]	398.5[b]	115.9[c]	24.1[b]	ND	31.3[b]	ND	1676.5[c]	55.4[b]	1699.2[c]	942.0[c]
	SU-SO	470.4[abc]	34.4[c]	347.3[b]	195.5[c]	0.0[b]	ND	0.0[b]	ND	1035.1[c]	0.0[b]	1098.9[c]	509.2[ab]
	SO	578.9[abc]	36.1[c]	427.7[b]	279.5[c]	7.3[b]	4.39[b]	6.3[b]	ND	1322.1[c]	18.1[b]	1340.2[c]	641.4[ab]
P values[2]		0.000	0.000	0.000	0.000	0.000	0.000	0.000	0.000	0.000	0.000	0.000	0.000
Physical refining (FAD, n=13)	LFAD	134.7	3.6	39.9	14.7[b]	90.0	ND	136.9	20.8	186.2	247.7	433.9	168.8
	PFAD	35.2	2.7	2.3	2.6[a]	21.5	17.5[b]	78.6	61.6	44.3	184.5	234.2	45.4
	OFAD	94.3	10.5	28.5	12.8[ab]	ND	ND	0.5	ND	145.9	0.5	146.4	101.4
P values[3]		0.514	0.076	0.094	0.024	0.231	0.017	0.423	0.152	0.241	0.429	0.676	0.514
P values[4]		0.000	0.000	0.000	0.026	0.220	0.000	0.125	0.000	0.000	0.065	0.000	0.000

Abbreviations: T, sum of α-, β-, γ-, and δ-tocopherol; T3, sum of α-, β-, γ- and δ-tocotrienol; T+T3, sum of tocopherols and tocotrienols; ND, not detected; SCP, blends of AO from seed oils, cocoa butter and palm oil; SP, blends of AO from seed and palm oils; O, AO from olive pomace oil and blends of AO from olive pomace and olive oils; BS, blends of AO from seed oils; SU, AO from sunflower oil; SU-SO, blends of AO from sunflower and soybean oils; SO, AO from soybean oil; LFAD, FAD from coconut oil and blends of FAD from coconut and palm kernel oils; PFAD, FAD from palm oil; OFAD, FAD from olive pomace and olive oils.

[1] The total vitamin E activity (expressed as mg of α-tocopherol/kg) was calculated using the activity conversion factors given by McLaughlin and Weihrauch (1979) for each T and T3.

[2] P values were obtained from Kruskal–Wallis U test and post-hoc comparisons for independent samples to compare medians between botanical origin groups within acid oils (AO, n=79). AO groups bearing different letters (a-c) are significantly different (P ≤0.05).

[3] P values were obtained from Kruskal–Wallis U test and post-hoc comparisons for independent samples to compare medians between botanical origin groups within fatty acid distillates (FAD, n=13). FAD groups bearing different letters (a-b) are significantly different (P ≤0.05).

[4] P values were obtained from Mann–Whitney U test for independent samples to compare medians between refining process (AO, n=79 vs FAD, n=13). P ≤0.05 was considered significant

Reference: Mclaughlin, P.J.; Weihrauch J.L. Vitamin E content of foods. J. Am. Diet Assoc. ***1979***, 75*, 647-665.*

Table S2. Median values of apparent metabolizable energy (AME, kcal/kg) for broilers and digestible energy (DE, kcal/kg) for pigs of different ages (young and adult) of acid oils and fatty acid distillates. As described in the Material and Methods section of the main article, AME and DE values were obtained according to Wiseman et al. (1998) equation and by applying the MIU (g/100g) correction to it as suggested by Bierinckx (2020).

		Wiseman et al. (1998) equation				Energy corrected by MIU (g/100g)			
		AME (broilers)		DE (pigs)		AME (broilers)		DE (pigs)	
		Young	Adult	Young	Adult	Young	Adult	Young	Adult
Chemical refining (AO, n=79)	SCP	5691.1^{a}	7141.9^{a}	7211.8^{a}	7440.4^{a}	5423.5^{a}	6806.1^{a}	6872.8^{a}	7090.6^{a}
	SP	6853.6^{b}	7776.4^{b}	7838.1^{b}	7968.3^{b}	6581.4^{b}	7476.0^{bc}	7555.5^{b}	7637.4^{b}
	O	7153.1^{c}	7941.0^{c}	7989.1^{c}	7972.9^{bc}	6610.0^{bc}	7336.8^{b}	7378.0^{b}	7385.6^{b}
	BS	7574.4^{d}	8221.7^{d}	8221.6^{d}	8169.2^{cd}	6813.7^{bcd}	7401.7^{bc}	7403.4^{b}	7363.6^{ab}
	SU	7621.7^{d}	8256.1^{d}	8244.5^{d}	8159.5^{d}	6872.1^{cd}	7483.5^{bc}	7487.1^{b}	7405.0^{b}
	SU-SO	7583.3^{d}	8227.6^{d}	8226.9^{d}	8168.0^{d}	6943.0^{d}	7634.5^{c}	7653.2^{b}	7589.5^{b}
	SO	7357.2^{cd}	8078.8^{cd}	8099.4^{cd}	8034.9^{bcd}	6827.8^{bcd}	7498.5^{bc}	7518.0^{b}	7458.8^{b}
P values[1]		0.000	0.000	0.000	0.000	0.000	0.000	0.000	0.000
Physical refining (FAD, n=13)	LFAD	5918.9^{b}	7244.5^{b}	7333.1^{b}	7555.3^{b}	5747.3^{b}	7045.1^{b}	7132.3^{b}	7350.3^{b}
	PFAD	4932.8^{a}	6683.3^{a}	6794.8^{a}	7013.8^{a}	4560.7^{a}	6216.8^{a}	6327.0^{a}	6530.0^{a}
	OFAD	7166.0^{b}	7968.0^{b}	7985.6^{b}	7835.5^{b}	6631.3^{b}	7374.5^{b}	7391.4^{b}	7253.3^{b}
P values[2]		0.006	0.006	0.006	0.006	0.006	0.006	0.006	0.008
P values[3]		0.000	0.000	0.000	0.000	0.000	0.000	0.000	0.000

See Table 1 in the main article for abbreviations.

[1] P values were obtained from Kruskal–Wallis U test and post-hoc comparisons for independent samples to compare medians between botanical origin groups within acid oils (AO, n=79). AO groups bearing different letters (a-d) are significantly different (P ≤0.05).

[2] P values were obtained from Kruskal–Wallis U test and post-hoc comparisons for independent samples to compare medians between botanical origin groups within fatty acid distillates (FAD, n=13). FAD groups bearing different letters (a-c) are significantly different (P ≤0.05).

[3] P values were obtained from Mann–Whitney U test for independent samples to compare medians between

References:

- *Wiseman, J.; Powles J.; Salvador, F. Comparison between pigs and poultry in the prediction of the dietary energy value of fats.* Anim. Feed Sci. Technol. ***1998***, 71*, 1-9. doi:* 10.1016/S0377-8401(97)00142-9

- *Bierinckx K. Variation in dietary lipids potentially influences your poultry production.* Int. Poult. Prod. ***2020***, 28*, 27-28. Available online:* http://www.positiveaction.info/pdfs/articles/pp28_3p27.pdf.

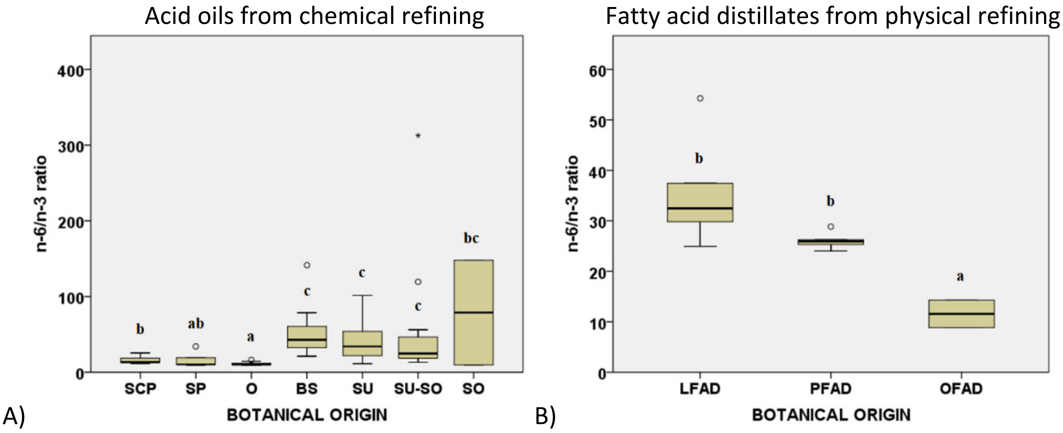

Figure S1. n-6 Polyunsaturated/n-3 polyunsaturated fatty acid ratio (n-6/n-3 ratio) boxplots according to botanical groups for acid oils from chemical refining (n= 79) and fatty acid distillates from physical refining (n=13). Within each type of refining, botanical groups bearing different letters (a-e) are significantly different according to Kruskal–Wallis test and post-hoc comparisons (P≤0.05). See Table S1 for botanical group abbreviations.

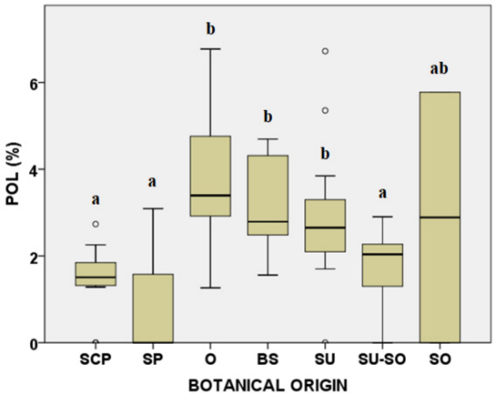

Figure S2. Polymeric compounds (POL) boxplots according to botanical groups for acid oils from chemical refining (n= 79). Botanical groups bearing different letters (a-b) are significantly different according to Kruskal–Wallis test and post-hoc comparisons (P≤0.05). See Table S1 for botanical group abbreviations. No POL were detected in FAD samples.

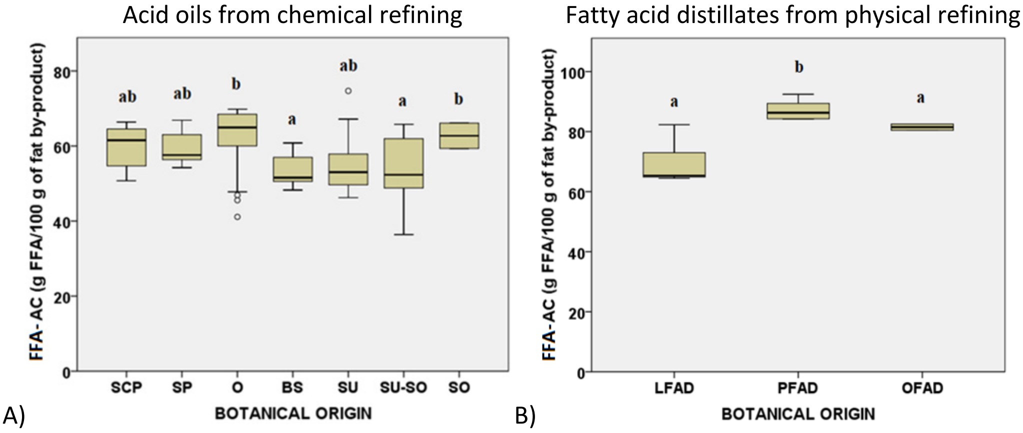

Figure S3. Acidity (FFA-AC, free fatty acids—FF—were expressed as lauric acid for the FAD coming from coconut and palm kernel oils, as palmitic acid for the palm FAD, and as oleic acid for the rest of samples) boxplots according to botanical groups for A) acid oils from chemical refining (n= 79) and B) fatty acid distillates from physical refining (n=13). Within each type of refining, botanical groups bearing different letters (a-b) are significantly different according to Kruskal–Wallis test and post-hoc comparisons (P≤0.05). See Table S1 for botanical group abbreviations.
